# Investigation of androgen receptor CAG repeats length in polycystic ovary syndrome diagnosed using the new international evidence-based guideline

**DOI:** 10.1186/s13048-023-01295-y

**Published:** 2023-11-07

**Authors:** Xueqi Yan, Xueying Gao, Qian Shang, Ziyi Yang, Yuteng Wang, Li Liu, Wei Liu, Dan Liu, Fang Cheng, Shigang Zhao, Han Zhao, Junli Zhao, Zi-Jiang Chen

**Affiliations:** 1 Center for Reproductive Medicine, Shandong University, Jinan, 250012 Shandong China; 2 State Key Laboratory of Reproductive Medicine and Offspring Health, Jinan, 250012 Shandong China; 3 Key Laboratory of Reproductive Endocrinology of Ministry of Education, Shandong University, Jinan, 250012 Shandong China; 4 Shandong Key Laboratory of Reproductive Medicine, Jinan, 250012 Shandong China; 5 Shandong Provincial Clinical Research Center for Reproductive Health, Jinan, 250012 Shandong China; 6 Shandong Technology Innovation Center for Reproductive Health, Jinan, 250012 Shandong China; 7 National Research Center for Assisted Reproductive Technology and Reproductive Genetics, Shandong University, Jinan, 250012 Shandong China; 8 Center for Reproductive Medicine, Ren Ji Hospital, School of Medicine, Shanghai Jiao Tong University, Shanghai, China; 9 Shanghai Key Laboratory for Assisted Reproduction and Reproductive Genetics, Shanghai, China; 10 Yinchuan Maternal and Child Health Hospital, Yinchuan, 750001 Ning Xia China; 11 Northwest Women’s and Children’s Hospital, Xi’an, Shanxi, 710100 China; 12 Department of Obstetrics and Gynecology, Reproductive Medicine Center, Tang Du Hospital, The Air Force Military Medical University, Xi’an, Shanxi, 710038 China; 13 General Hospital of Ningxia Medical University, Yinchuan, China; 14 Department of Reproductive Medicine, General Hospital of Ningxia Medical University, Ningxia, China

**Keywords:** Polycystic ovary syndrome, Polycystic ovarian morphology, Androgen receptor, CAG repeats, Genetic polymorphism

## Abstract

**Background:**

To study whether CAG repeat polymorphism of androgen receptor (*AR*) contributes to the risk of polycystic ovarian morphology (PCOM) with antral follicle count (AFC) ≥ 20 in the context of new international guideline of polycystic ovary syndrome (PCOS).

**Methods:**

Blood of 109 PCOS cases and 61 controls were collected for the measurement of AR CAG repeats length by sequencing. The mean number and frequency distribution of CAG repeats length were observed. Detailed analysis was conducted by dividing PCOS cases into low AFC group (L-AFC, AFC < 20) and high AFC group (H-AFC, AFC ≥ 20) according to the new international evidence-based guideline.

**Results:**

The portion of individuals with lower CAG repeats length in H-AFC group was significantly larger than those with higher CAG repeats length. Logistic model revealed individuals with lower CAG length tended to develop H-AFC.

**Conclusion:**

Lower CAG repeats length in the *AR* gene of PCOS cases increases risk of PCOM.

**Supplementary Information:**

The online version contains supplementary material available at 10.1186/s13048-023-01295-y.

## Background

Polycystic ovary syndrome (PCOS) is a prevalent endocrine and metabolic disorder in women of childbearing age, with a prevalence of 7.8% [1]. PCOS is characterized by a series of reproductive abnormalities, including of oligo-/ano-ovulation, hyperandrogenism (HA) and polycystic ovarian morphology (PCOM) [2]. As the key feature of PCOS, HA results from abnormal gonadotropin releasing hormone stimulating ovarian theca cell to produce androgen. The hormone environment impedes follicular maturation, resulting in a considerable amount of small antral follicles and ovulatory dysregulation [3, 4].

Androgen receptor (*AR*) is one of PCOS candidate genes [5, 6]. *AR* belongs to a nuclear receptor superfamily of transcription factors and locates on Xq11-12 [7]. It has three major domains: an N-terminal transactivation domain, a DNA-binding domain and a C-terminal hormone-binding domain [8]. The N-terminal transactivation domain is encoded by the exon one of *AR* gene and is composed of a polyglutamine tract, which is encoded by a variable length of CAG repeat polymorphism. Due to the two alleles of X chromosome in females, one allele with relatively longer CAG repeat length is termed as “long allele” and another allele is termed as “short allele”. “Biallelic average” is equal to the average length of “short allele” and “long allele”. In recent years, several studies concentrated on the relationship between CAG repeat polymorphism and PCOS [9–11].

Simultaneously, *AR* is highly expressed in granulosa cells of pre- and early antral follicles and decreases during follicular maturation, indicating that *AR* plays an important role in follicular development [12–14]. The new international PCOS guideline recommends antral follicle count (AFC) ≥ 20 as a new standard of PCOM [15]. Based on the new cutoff of PCOM, our previous study divided PCOS into low AFC (L-AFC) group and high AFC (H-AFC) group and reported that the *AR* expression decreased significantly in PCOS, especially for the H-AFC group [16].

However, the relationship between *AR* CAG polymorphism and PCOM based on the new PCOS guideline has not been investigated yet. Hence, we conducted this study to investigate the relationship between PCOM and CAG repeats length.

## Methods

### Study population

A total of 170 participants with PCOS cases and controls aged 18–45 years were recruited from the reproductive center of General Hospital of Ningxia Medical University. All available details (age, weight and height) were recorded. PCOS cases were included according to the Rotterdam Revised 2003 diagnosis criteria [17]: oligo-/ano-ovulatory, clinical or biochemical hyperandrogenism (HA) and PCOM diagnosed by transvaginal ultrasound. The diagnosis can be defined when two of three are fulfilled. Clinical HA included hirsutism, acne and so on. Individuals with total testosterone concentration above 48.1 ng/dL will be defined with biochemical HA. The exclusion criteria consisted of androgen tumor, congenital adrenal hyperplasia, Cushing’s syndrome, thyroid related disease and so on. And the controls were who visited the clinic due to oviduct dysfunction or male infertility and all of them had normal menstrual cycle and ovarian morphology.

### *AR* CAG length measurement and analysis

DNA was isolated from peripheral blood by TIANamp Genomic DNA Kit (TIANGEN, China) protocol and quantified by spectrophotometry. Primers of *AR* were constructed in Shanghai Generay Biotechnology: fluorescent-labeled forward primer FAM-5’-TCCAGAATCTGTTCCAGAGCGTGC-3’ and reverse primer 5’-GCTGTGAAGGTTGCTGTTCCTCAT-3’. According to previous reported method [18], the genomic DNA was amplified by polymerase chain reaction (PCR) with *AR* primers. PCR products were sequenced by ABI 3730 DNA Sequencer (Applied Biosystems, USA) under standard conditions and analyzed by Peak Scanner software to determine the length genetic polymorphism.

As the *AR* gene is located on the X chromosome and two alleles exist in women, one allele with relatively longer CAG repeat length was termed as “long allele” and another allele was termed as “short allele”. We employed the conventional method to analyze the alleles: 1) the mean value of the two alleles (biallelic average), 2) the short allele alone, and 3) the long allele alone. The median values of CAG repeats length are 22.5 for biallelic average, 21 for short allele and 24 for long allele, which were used as the cutoff to divide CAG repeats length into lower and higher part for the frequency distribution analysis.

### Clinical and biochemical measurement

For individuals with normal cycle, peripheral blood was collected on the day 3 of menstrual cycle; for women with oligo-/amenorrhea, peripheral blood was collected at any time. The serum follicle stimulating hormone, luteinizing hormone, estradiol and total testosterone were measured using chemiluminescence immunoassay and enzyme-linked immunosorbent assay in the clinical laboratory of Reproductive center of Ningxia Medical university. Transvaginal ultrasound was used to evaluate the AFC in the follicular stage.

### Statistical analysis

Variables corresponding to normal distribution were compared with independent t-test between controls and PCOS. And the non-normal variables were analyzed with non-parametric Mann–Whitney U-test between controls and PCOS. In the subgroup analysis, one-factor analysis of variance test and least significance difference were used to compare different data. Chi-square distribution (χ^2^) test was used to compare the distribution frequency among different groups. Logistic regression model was constructed with the presence or absence of long allele < 24 and biallelic average < 22.5 as the independent variable and the presence or absence of PCOS/L-AFC/H-AFC as the dependent variable separately. All the statistical analysis were performed with the SPSS version 26. Statistical significance was defined as a two-side p value less than 0.05, and data was reported as mean ± SD and number (percent).

## Results

### The *AR* CAG repeats length in PCOS cases and controls

The baseline information of controls and PCOS cases were listed in Table 1.

**Table 1:** The clinical features and AR CAG frequency distribution of controls and PCOS cases

Variable		Control (*n* = 61)	PCOS (*n* = 109)	*p* value
Baseline				
Age (years)		29.52 ± 4.43	27.59 ± 4.38	0.006
BMI (kg/m^2^)		23.15 ± 3.57	25.32 ± 4.20	0.001
LH (IU/L)		3.57 ± 1.67	10.00 ± 6.19	< 0.001
FSH (IU/L)		5.93 ± 2.19	5.86 ± 1.66	0.837
E_2_ (pg/mL)		46.69 ± 24.30	56.03 ± 37.99	0.116
TT (ng/dL)		41.76 ± 15.94	65.98 ± 34.63	< 0.001
AR CAG frequency distribution^a^				
Short allele	< 21	17(27.87%)	40(36.70%)	0.242
	≥ 21	44(72.13%)	69(63.30%)	-
Long allele	< 24	24(39.34%)	40(36.70%)	0.733
	≥ 24	37(60.66%)	69(63.30%)	-
Biallelic average	< 22.5	20(32.79%)	46(42.20%)	0.277
	≥ 22.5	41(67.21%)	63(57.80%)	-

All values were reported as mean ± SD and number (percent)

*Abbreviations*: *PCOS* polycystic ovary syndrome, *BMI* body mass index, *LH* luteinizing hormone, *FSH* follicle stimulating hormone, *E*_*2*_ estradiol, *TT* total testosterone, *AR* androgen receptor

*P* value was given by the independent t-test and chi-square test

^a^represents the median CAG repeats length of all cases including controls and PCOS

**Fig. 1:**
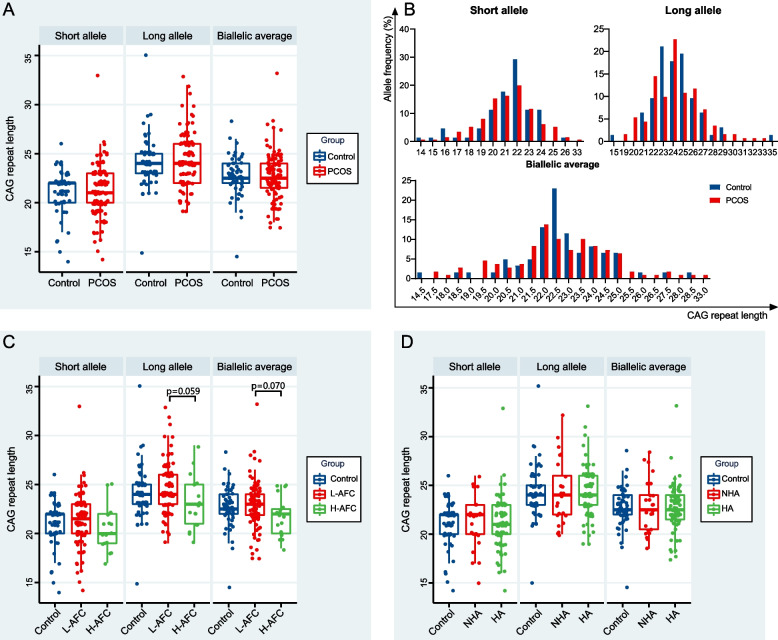
**A** CAG repeats length of *AR* gene in controls and polycystic ovary syndrome (PCOS). **B** CAG allele distribution. Frequency distribution of CAG allele in exon one of *AR* gene in PCOS cases and controls for short allele, long allele and biallelic average. The blue band represented controls and the red band represented PCOS cases. **C** CAG repeats length of *AR* gene in controls, L-AFC and H-AFC PCOS cases. P represented *p* value given by the least significance difference. L-AFC, low AFC (AFC < 20); H-AFC, high AFC (AFC ≥ 20). **D** CAG repeats length in *AR* gene in control, NHA and HA PCOS cases. NHA, non-hyperandrogenism; HA, hyperandrogenism

No difference was found in the mean CAG repeats length between PCOS cases and controls, whether using short allele, long allele or biallelic average of them (Fig. 1A).

Allele distribution was also similar between the two groups (Fig. 1B). *AR* CAG repeats length ranged from 14–33 among PCOS cases, and from 14–35 among controls. Frequency distribution analysis showed us no difference of frequency distribution between PCOS cases and controls (Table 1).

### The *AR* gene CAG repeats length in low and high AFC group

To further investigate the relationship between PCOM and CAG repeat polymorphism, PCOS group was categorized into L-AFC and H-AFC group. The clinical manifestation of controls, L-AFC and H-AFC PCOS cases was presented in Table 2.

**Table 2:** The clinical features and AR CAG frequency distribution of controls, L-AFC and H-AFC PCOS cases

Variable		Control (*n* = 61)	L-AFC (*n* = 92)	H-AFC (*n* = 17)	*p* value^**a**^ (overall)	*p* value^**b**^ (L-AFC vs H-AFC)
Baseline						
Age (years)		29.52 ± 4.43	27.68 ± 4.61	27.06 ± 2.86	0.021	0.591
BMI (kg/m^2^)		23.15 ± 3.57	25.11 ± 3.98	26.45 ± 5.25	0.002	0.205
LH (IU/L)		3.57 ± 1.67	9.86 ± 6.29	10.71 ± 5.80	< 0.001	0.547
FSH (IU/L)		5.93 ± 2.19	5.87 ± 1.53	5.79 ± 2.26	0.965	0.868
E_2_ (pg/mL)		46.69 ± 24.30	54.72 ± 37.07	62.73 ± 43.01	0.854	0.207
TT (ng/dL)		41.76 ± 15.94	67.07 ± 36.29	60.55 ± 24.88	< 0.001	0.607
AR CAG frequency distribution*						
Short allele	< 21	17(27.87%)	31(33.70%)	9(52.94%)	0.153	0.130
	≥ 21	44(72.13%)	61(66.30%)	8(47.06%)	-	-
Long allele	< 24	24(39.34%)	30(32.61%)	10(58.82%)	0.115	0.039
	≥ 24	37(60.66%)	62(67.39%)	7(41.18%)	-	-
Biallelic average	< 22.5	20(32.79%)	35(38.04%)	11(64.71%)	0.056	0.041
	≥ 22.5	41(67.21%)	57(61.96%)	6(35.29%)	-	-

All values were reported as mean ± SD and number (percent)

*Abbreviations*: *PCOS* polycystic ovary syndrome, *AFC* antral follicle count, *BMI* body mass index, *LH* luteinizing hormone, *FSH* follicle stimulating hormone, *E2* estradiol, *TT* total testosterone, *AR* androgen receptor, *L-AFC* low AFC (AFC < 20), *H-AFC* high AFC (AFC ≥ 20)

^*^Represents the median CAG repeats length of all cases including controls and PCOS

^a^*P* value was given by the one-factor analysis of variance and chi-square test among three groups

^b^*P* value was given by the post-hoc analysis of least significance difference and chi-square test between L-AFC and H-AFC groups

Examination of the mean CAG repeats length revealed that the *AR* CAG repeats length in the H-AFC group was lower than in the L-AFC group (Fig. 1C).

For the allele distribution, we found that most individuals in H-AFC group had lower CAG repeats lengths than those in L-AFC group (Table 2). Results showed that 58.82% of H-AFC group for long allele and 64.71% of H-AFC group for biallelic averages had lower CAG repeats lengths (less than 24 for long allele and 22.5 for biallelic average). Frequency comparison between L-AFC and H-AFC group showed us significant difference (*p* = 0.039 for long allele and *p* = 0.041 for biallelic averages).

### The *AR* gene CAG repeats length in non-hyperandrogenism (NHA) PCOS and hyperandrogenism (HA) PCOS

As the *AR* gene CAG repeats length is correlated with the action of *AR*, we further divided the PCOS group into NHA and HA subgroup with clinical features listed in Supplemental Table 1. No difference of mean number for short allele, long allele and biallelic average was observed among controls, NHA-PCOS and HA-PCOS (Fig. 1D). In addition to that, there was also no difference on the frequency distribution in the PCOS subgroup (Supplemental Table 2).

### Lower CAG repeat length contributes to the risk of H-AFC in PCOS

Given the significant difference of CAG allele distribution frequency between L-AFC and H-AFC, we constructed the binary logistic regression model to determine whether lower CAG repeat length contributes to the risk of PCOS or PCOM. The results showed us that there is no relationship between lower CAG repeats length and PCOS (Table 3).

**Table 3:** Predictive models of PCOS and subgroups with CAG biallelic average

Model	Variable	PCOS VS Control		**L-AFC VS Control**		**H-AFC VS Control**		H-AFC VS L-AFC	
		OR	*p* value	OR	*p* value	OR	*p* value	OR	*p* value
1	Long allele < 24	0.894	0.733	0.746	0.394	2.202	0.157	2.952	0.045
2	Long allele < 24	0.803	0.532	0.693	0.316	1.694	0.387	2.996	0.048
	Age	0.896	0.005	0.907	0.013	0.826	0.037	0.945	0.390
	BMI	1.180	0.001	1.171	0.002	1.204	0.013	1.064	0.318
3	Biallelic average < 22.5	1.497	0.228	1.259	0.507	3.758	0.022	2.986	0.047
4	Biallelic average < 22.5	1.315	0.439	1.158	0.687	3.112	0.069	2.827	0.062
	Age	0.897	0.006	0.906	0.012	0.827	0.047	0.966	0.590
	BMI	1.173	0.001	1.164	0.003	1.202	0.017	1.066	0.299

These models included the presence or absence of long allele < 24 and biallelic average < 22.5 as the independent variable and the presence or absence of PCOS/L-AFC/H-AFC as the dependent variable separately

*Abbreviations*: *PCOS* polycystic ovary syndrome, *BMI* body mass index, *AFC* antral follicle count, *L-AFC* low AFC (AFC < 20), *H-AFC* high AFC (AFC ≥ 20)

Furthermore, we investigated whether CAG repeats length contributed to the risk of PCOS subgroup. The logistic regression analysis indicated that long allele < 24 was associated the incidence of H-AFC compared with L-AFC PCOS (Table 3). For PCOS women with CAG < 24, the risk to develop H-AFC was more than twice times larger than those with CAG ≥ 24. Additionally, the results showed that biallelic average < 22.5 contributed to the risk of PCOS with H-AFC compared with control, however, the significance disappeared after the adjustment of age and BMI. There was no relationship between CAG repeats length and the risk of PCOS with L-AFC, indicating that the CAG repeats length was primarily associated with the risk of developing H-AFC in PCOS (Table 3).

## Discussion

In the present study, the CAG length of our participants ranged from 14–35 in total, which is in the normal range [19]. According to our data, we found no significant difference in the mean CAG repeats length between PCOS cases and controls, which was consistent with previous studies [10, 19–22]. Apart from this, the frequency distribution of CAG repeats length was found no distinction between PCOS and controls. However, some studies found that PCOS exhibited a greater frequency of CAG repeats length longer than 22 repeats [9], while some studies presented that individuals with precocious pubarche had greater proportion of short allele less than 22 repeats [23]. For these inconsistent results, further studies involving a larger number of women are needed.

As for the AFC, we split PCOS group into L-AFC and H-AFC subgroup according to the new PCOS guideline. The H-AFC group had a lower CAG repeats length than the L-AFC group. Moreover, a significantly greater proportion of the H-AFC group had CAG repeats length less than 24 for long allele or less than 22.5 for biallelic averages. Logistic regression analysis suggested individuals with CAG < 24 for long allele were more likely to be affected by PCOM.

Disordered follicle development is regulated by the interaction of androgen and AR [13, 24, 25]. In our cohort, the total testosterone concentration in H-AFC group was lower than L-AFC group. It has been reported that the shorter CAG length in H-AFC can increase AR sensitivity to androgen [7, 26]. Therefore, lower CAG repeats length in H-AFC group contributes to increase transactivation of AR, resulting in the incidence of follicular arrest and an excessive number of small antral follicles.

Our previous study also investigated the relationship between *AR* and PCOM with cutoff of new guideline, which concentrated on the *AR* expression of granulosa cells and PCOM. Those results suggested decreased *AR* expression in PCOS group, especially in the H-AFC group [16]. The inconsistency of results may result from the different tissues, this means that peripheral blood was used in the present study, while granulocytes were used in the previous study. Despite differences, the commonality of them suggested that *AR* is different between H-AFC and L-AFC group in PCOS, no matter from its expression or its CAG polymorphism.

The study gave insight into the association between *AR* CAG polymorphism and PCOM diagnosed based on the new international guideline, demonstrating that CAG polymorphism had an influence on the risk of H-AFC in PCOS. Whereas, the sample size, particularly in the H-AFC subgroup, was relatively small. Further investigation is needed in a larger population and also for the specific mechanism.

In conclusion, we enrolled 61 controls and 109 PCOS cases in the General Hospital of Ningxia Medical University and tested the hormonal parameter and *AR* CAG repeats length. Our results showed no significant difference in mean CAG repeats length and distribution frequency between controls and PCOS cases or between HA and NHA PCOS. However, our results revealed that individuals in the H-AFC group had a shorter mean CAG repeats length and a larger fraction of H-AFC group tends to have shorter CAG length for biallelic averages and long allele. Logistic regression model suggested that CAG < 24 for long allele can increase the risk of H-AFC in PCOS.

The exact diagnosis of PCOS, a common reproductive-age disease with substantial health and economic burden, is important for our society [27]. The new international guideline for PCOS aims to reduce the overdiagnosis and provide more accurate diagnosis. In the direction of new guideline, our study adds to the accumulating evidence that AR signaling plays an important role in the follicular development and provides insight on the relationship between CAG polymorphism and follicular arrest.

## Conclusions

In this study, we demonstrated that the portion of individuals with lower CAG repeats length in H-AFC group was significantly larger than those with higher CAG repeats length. Logistic model revealed that individuals with lower CAG length tended to develop H-AFC, suggesting that CAG repeats length contributed to the risk of PCOM in the setting of new international PCOS guideline.

### Supplementary Information

**Additional file 1: Supplemental Table 1.** Clinical features of controls, NHA and HA PCOS.

**Additional file 2: Supplemental Table 2.** Frequency distribution of CAG length in controls, NHA and HA-PCOS.

## Data Availability

The datasets generated and/or analyzed during the current study are not publicly available but are available from the corresponding author on reasonable request.

## Abbreviations

AFC: Antral follicle count
AR: Androgen receptor
HA: Hyperandrogenism
H-AFC: High antral follicle count
L-AFC: Low antral follicle count
NHA: Non-hyperandrogenism
PCOM: Polycystic ovarian morphology
PCOS: Polycystic ovary syndrome
PCR: Polymerase chain reaction
