# Supplementary material for: Investigation of androgen receptor CAG repeats length in polycystic ovary syndrome diagnosed using the new international evidence-based guideline
Source: J Ovarian Res. 2023 Nov 7;16:211. doi: 10.1186/s13048-023-01295-y (PMC10629046; doi:10.1186/s13048-023-01295-y)
Supplement: Supplementary file 1 — Additional file 1: Supplemental Table 1. Clinical features of controls, NHA and HA PCOS. [file 13048_2023_1295_MOESM1_ESM.docx]

**Supplemental Table 1: clinical features of controls, NHA and HA PCOS.**

| Clinical parameters | Control  (n=61) | NHA-PCOS (n=25) | HA-PCOS (n=77) | P value |
| --- | --- | --- | --- | --- |
| Age (years) | 29.52±4.43 | 29.04±5.08 | 27.27±4.17 | 0.010 |
| BMI (kg/m^2^) | 23.15±3.57 | 25.41±4.52 | 25.44±4.24 | 0.003 |
| LH (IU/L) | 3.57±1.67 | 5.70±3.84 | 11.37±6.31 | ＜0.001 |
| FSH (IU/L) | 5.93±2.19 | 5.52±1.70 | 5.95±1.67 | 0.577 |
| E_2_ (pg/mL) | 46.69±24.30 | 40.44±22.13 | 57.38±28.33 | ＜0.001 |
| TT (ng/dL) | 41.76±15.94 | 32.74±16.85 | 76.78±31.98 | ＜0.001 |

All values were reported as mean±SD. P value was given by the one-factor analysis of variance.

Abbreviations: PCOS, polycystic ovary syndrome; NHA, non-hyperandrogenism; HA, hyperandrogenism; BMI, body mass index; LH, luteinizing hormone; FSH, follicle stimulating hormone; E2, estradiol; TT, total testosterone.
