# Supplementary material for: Investigation of androgen receptor CAG repeats length in polycystic ovary syndrome diagnosed using the new international evidence-based guideline
Source: J Ovarian Res. 2023 Nov 7;16:211. doi: 10.1186/s13048-023-01295-y (PMC10629046; doi:10.1186/s13048-023-01295-y)
Supplement: Supplementary file 2 — Additional file 2: Supplemental Table 2. Frequency distribution of CAG length in controls, NHA and HA-PCOS. [file 13048_2023_1295_MOESM2_ESM.docx]

**Supplemental Table 2: frequency distribution of CAG length in controls, NHA and HA-PCOS.**

|  | CAG repeats length^*^ | NHA | HA | p value |
| --- | --- | --- | --- | --- |
| Short allele | ＜21 | 10(40.00%) | 28(36.36%) | 0.777 |
|  | ≥21 | 15(60.00%) | 49(63.64%) |  |
| Long allele | ＜24 | 12(48.00%) | 28(36.36%) | 0.300 |
|  | ≥24 | 13(52.00%) | 49(63.64%) |  |
| Biallelic average | ＜22.5 | 12(48.00%) | 33(42.86%) | 0.653 |
|  | ≥22.5 | 13(52.00%) | 44(57.14%) |  |

Comparison of categorial variable between groups was done by chi-square test.

*Represents the median CAG repeats length of controls and PCOS cases.

Abbreviations: PCOS, polycystic ovary syndrome; NHA, non-hyperandrogenism; HA, hyperandrogenism.
